# The Mutation V42M Distorts the Compact Packing of the Human Gamma-S-Crystallin Molecule, Resulting in Congenital Cataract

**DOI:** 10.1371/journal.pone.0051401

**Published:** 2012-12-21

**Authors:** Venkata Pulla Rao Vendra, Sushil Chandani, Dorairajan Balasubramanian

**Affiliations:** 1 Prof. Brien Holden Eye Research Centre, Hyderabad Eye Research Foundation, L. V. Prasad Eye Institute, Hyderabad, India; 2 Novarus Discoveries Pvt Ltd, Hyderabad, India; Semmelweis University, Hungary

## Abstract

**Background:**

Human γS-crystallin is an important component of the human eye lens nucleus and cortex. The mutation V42M in the molecule causes severe congenital cataract in children. We compare the structure of the mutant protein with that of the wild type in order to understand how structural changes in the mutant relate to the mechanism of opacification.

**Methods:**

Both proteins were made using conventional cloning and expression procedures. Secondary and tertiary structural features of the proteins were analyzed using spectral methods. Structural stabilities of the proteins were analyzed using chemical and thermal denaturation methods. Self-aggregation was monitored using extrinsic spectral probes. Molecular modeling was used to compare the structural features of the two proteins.

**Results:**

While the wild type and mutant have the same secondary structure, molecular modeling and fluorescence analysis suggest the mutant to have a more open tertiary structure, with a larger hydrophobic surface. Experiments using extrinsic probes reveal that the mutant readily self-aggregates, with the suggestion that the aggregates might be similar to amyloidogenic fibrils. Chemical denaturation indicates that while the wild type exhibits the classic two-state transition, V42M goes through an intermediate state, and has a distinctly lower stability than the wild type. The temperature of thermal unfolding of the mutant is also distinctly lower. Further, the mutant readily precipitates and scatters light more easily than the wild type.

**Conclusion:**

The replacement of valine in position 42 by the longer and bulkier methionine in human γS-crystallin perturbs the compact β-sheet core packing topology in the N-terminal domain of the molecule, exposes nonpolar residues thereby increasing the surface hydrophobicity and weakens the stability of the protein, thus promoting self-aggregation leading to light scattering particles. This set of changes in the properties of the mutant offers a molecular insight into the mechanism of opacification.

## Introduction

Cataract, or the opacification of the eye lens, is the leading cause of blindness the world over. It is known to be caused by a variety of metabolic disorders which are either inherited or acquired over time. While a variety of factors lead to lens opacity, perhaps the least complicated and single factor etiology is seen in congenital cataracts, occurring at birth, which are the result of genes expressed in the lens.

Mutations over 23 genes have been reported to be associated with congenital cataracts [1]–[3]. Of these, the major genes are those of the crystallins. The transparency and high refractive index of the human eye lens depends on the stability and solubility of crystallins, a class of cytosolic structural proteins that constitute 95% of the water- soluble proteins, contributing to about 35% of the lens mass. Of the three groups of crystallins in the human lens, two members of the α-family αA and αB, account for 40%, seven of the β-family (βA1, βA2, βA3, βA4, βB1, βB2 and βB3) for about 35% and three γ crystallins (γC, γD and γS) about 25% of the total crystallin content. The monomer molecular weight of each of these crystallins is between 19–27 kD. α-Crystallins form multimeric aggregates 800–1200 kD in size, β-crystallins occur as oligomers (dimers to octamers), while γ-crystallins exist as monomers. The human γ-crystallin gene cluster has γA, γB, γC, γD, γS and a fragment of γG, each with 3 exons, as members; however, only γC, γD and γS-crystallins encode abundant lens γ-crystallins in humans [4]. The abundance of the crystallins within the human lens is asymmetric and biphasic; the lens fiber cells, which make up the nuclear region and the bulk of the lens are richer in β- and γ-crystallins than the cortex and epithelium [5].

The crystal structures of several β and γ crystallins have been determined and they are found to form a superfamily defined by the βγ-crystallin fold a double domain structure containing four runs of highly stable “Greek key” motifs. Each domain of the β- and γ-crystallins consists of two intercalated antiparallel β-sheet Greek key motifs. It has been reported that the high stabilities of the β- and γ-crystallin proteins are due to the compact topology of the double Greek key [6]. The crystal structure of the C-terminal domain of human γS crystallin (HGSC) has been studied [7] and the solution structure of the full length murine γS crystallin has been resolved by NMR methods [8].

It thus becomes possible to go beyond reporting the mutations in crystallins associated with congenital cataract and to study the functional genetics, or attempt a protein structural rationale. We chose to study HGSC here, since it is richly expressed in the cortical cells and seen in the nuclear region, and four mutations in this protein (G18V, D26G, S39C and V42M) have so far been reported to be associated with congenital cataracts [9], [10], [11], [12]. Studying their structure should provide the link to the mechanism of opacification. Analysis of the structure and stability of the wild type and G18V mutant proteins in human has been done [13], [14] and the F9S mutation in the mouse γS gene associated with *Opj* cataract in mice has also been studied [15], [16], [17]. In this present study, we attempt to understand the structural properties of the HGSC mutant V42M. A mis-sense mutation of γS gene, c.176G→A, which results in the replacement of a highly conserved valine by methionine at position 42 in the protein, has been reported to co-segregate with bilateral congenital cataract [12]. We show that this single point mutation loosens up the compact packing of the molecule, exposing nonpolar residues to the surface and weakening the stability, causing self-aggregation leading to light-scattering particles.

## Materials and Methods

### Cloning

Wild type γS cDNA was cloned into pET-21-a vector and its mutant pET-21-a-γSV42M was generated using the procedure previously described [18], [19]. The sequences were checked prior to overexpression using ABI 3130 genetic analyzer and found to be correct. The primers used for cloning and sequencing are listed in Table 1.

**Table 1:** List of primers used for cloning and sequencing.

Clone	Primer	Primer sequence
pET21-a-γS	Forward	5′GGGAGTTCCATATGTCTAAAACTGGAACC3′
pET21-a-γS	Reverse	5′CCGGAATTCTTACTCCACAATGCG3′
pET21-a-γSV42M	Forward	5′GCTGCAACTCCATTAAAATGGAAGGAGGCACCTGGGCTG3′
pET21-a-γSV42M	Reverse	5′CCAGGTGCCTCCTTCCATTTTAATGGAGTTGCAGCGACTTAGG3′
T7	Forward	5′TAATACGACTCACTATAGG3′
T7	Reverse	5′TATGCTAGTTATTGCTCAG3′
BGH	Reverse	5′TAGAAGGCACAGTCGAGG3′

### Overexpression of Recombinant Proteins

The recombinant constructs pET21-a-γS wild type and pET21-a-γSV42M were transformed into E. coli BL21(DE3) pLys cells. A single colony containing the pET21-a-recombinant plasmid was picked, inoculated into 15 ml of Luria-Bertoni (LB) medium containing 50 µg/ml ampicillin and 34 µg/ml chloramphenicol and grown for 12 h by shaking at 225 rpm at 37°C. After 12 h, 10 ml of culture was transferred into 1L of the above medium. The cultures were grown at 37°C to an absorbance value of 0.6 at 600 nm. Recombinant protein synthesis was induced by the addition of isopropyl-1-thio-D-galactopyranoside (IPTG) to a final concentration of 1 mM and the cultures were grown for an additional 3.5 hr. Cells were pelleted down from the 1L culture by centrifugation at 6000 *g* for 10 min at 4°C.

The pellets were suspended in 40 mL of Lysis Buffer (composition: 50 mM sodium acetate (pH 4.75), 1 mM dithiothreitol (DTT), 1 mM phenylmethylsulfonyl fluoride (PMSF) and 20 µg/ml aprotinin). The cell suspension was extensively sonicated for 40 cycles (15 s each cycle) with 45 s intervals at 35% amplitude at 4°C using a high intensity ultrasonic processor (Sonics Vibra Cell, Sonics & Materials Inc, Newton, MA).The cell lysate was centrifuged at 30,000 *g* for 20 min at 4°C. The supernatant and the pellet were checked for the presence of the protein on 14% SDS-PAGE. Wild type and V42M were predominantly found in the soluble fraction, and hence we chose the supernatant as the source of the target protein.

### Purification of WT and Mutant Proteins

The supernatant was chromatographed using a SP-Sepharose ion-exchange column. The column was equilibrated with 50 mM sodium acetate buffer (pH 4.75) and the supernatant was loaded on to the column and eluted using a salt gradient of 0–1 M KCl. The fractions containing the required protein were pooled, concentrated using an Amicon stirred ultrafiltration cell with a 3 kD cut-off membrane and further purified to homogeneity on a Sephadex G-75 column. The purity of the proteins was checked by SDS-PAGE and Western blotting. The concentrations of each protein was calculated using the extinction coefficient of HGSC at 280 nm = 41,040 cm^−1^ M^−1^, as per Mills et al. [20].

### Spectroscopic Analysis

Circular dichroism (CD) spectra were recorded using a dichroigraph instrument (J-810; Jasco, Easton, MD) at room temperature (27°C). Far-ultraviolet CD spectra (region 250–193 nm) were recorded with 2 mm path length quartz cells and the near-UV CD spectra (320–250 nm) were recorded with 1 cm path length quartz cells, with 2 s response time at 100 nm/s speed. Three scans of each spectrum were averaged, and baselines of the buffer alone were subtracted. The protein concentration used for determining the far-UV spectra was 10 µM (0.2 mg/ml) in 10 mM sodium phosphate buffer (pH 7.0) and for the near-UV spectra, it was 40 µM (0.8 mg/ml) in 100 mM sodium phosphate buffer (pH 7.0).

Intrinsic fluorescence spectra were recorded at room temperature (27°C) using a fluorescence spectrophotometer (F-2500; Hitachi, Yokohama, Japan) and the spectra were recorded in the range of 300 to 400 nm using an excitation wavelength of 295 nm, with 2.5 nm excitation and emission slits. The protein concentrations used were 10 µM (0.2 mg/ml) in 100 mM sodium phosphate buffer (pH 7.0). Three scans of each spectrum were averaged, and baselines of the buffer alone were subtracted. Quenching experiments were done using KI and acrylamide as quenchers, and the results were analyzed using the Stern-Volmer approach, following Augusteyn et al [21].

Extrinsic fluorescence spectra of proteins were recorded using two surface hydrophobicity probes, namely 4,4′-dianilino-1,1′-binaphthyl-5,5′-disulfonate (bis-ANS) [22] and 9-diethylamino-5H-benzo[alpha]phenoxazin-5-one (Nile Red) [23]. With bis-ANS, spectra were recorded in the range of 400 to 600 nm, using an excitation wavelength of 390 nm with 2.5 nm excitation and emission slits. With Nile Red, the excitation was at 540 nm and the emission recorded between 570 and 700 nm, using 10 nm excitation and emission slits slits. The protein concentration used in each case was 5 µM (0.1 mg/ml) in 100 mM sodium phosphate buffer (pH 7.0). Stock solutions of bis-ANS and Nile Red were prepared in methanol and the final alcohol concentration was maintained below 7% (v/v) when the reagents were mixed with the proteins. Concentrations of bis-ANS and Nile Red were measured using extinction coefficients of 16.8 mM^−1^ cm^−1^ at 385 nm and 45 mM^−1^ cm^−1^ at 552 nm respectively. We also made use of the fluorescence of the terbium ion (Tb^3+^), which acts as an excellent Ca^2+^-mimic, with emission bands near 490 nm and 546 nm, upon excitation at 285 nm (through an energy transfer mechanism from the protein), when bound to the Ca^2+^binding sites in proteins [24], [25].

### Monitoring Amyloid-type Behavior

Formation of amyloid-type fibrils was monitored at room temperature (27°C) using the probe thioflavin-T [26]. Spectra were recorded in the range of 470 to 570 nm, using an excitation wavelength of 444 nm with 10 nm excitation and emission slits. Protein concentrations used in each case were 5 µM (0.1 mg/ml) in 100 mM sodium phosphate buffer (pH 7.0). Baselines of the buffer alone were subtracted.

### Protein Unfolding Induced by Guanidine Hydrochloride

Equilibrium unfolding and refolding experiments were performed at room temperature (27°C) by diluting the purified proteins to 0.2 mg/ml in a series of solutions ranging in concentrations of 0 to 4.5 M guanidine hydrochloride (GuHCl) in a buffer containing 100 mM sodium phosphate, 1 mM EDTA and 5 mM DTT. Samples were incubated at 37°C for 16 hrs. The procedure used here was the same as that of Mills et al. [20]. Fluorescence emission spectra were recorded for each unfolding sample using a spectrofluorimeter as described above. Data were analyzed by plotting the concentration of GuHCl for each sample versus the ratio of fluorescence intensities at 360 and 320 nm. Equilibrium unfolding data were fit to the two-state model of Greene and Pace [27], or the three-state model of Clark et al. [28], using Graphpad prism software. The model that best fit the data was selected based on a random distribution of residuals. Transition midpoints and ΔG^0^ were calculated for all transitions from these fits. In all fluorescence experiments, the response time used was 0.08 s, scan speed 60 nm/s and the PMT voltage was below 400V.

### Thermal Denaturation Followed by Calorimetry

Differential scanning calorimetry was done using a VP-DSC Micro Calorimeter instrument (VP-DSC, Piscataway, NJ, USA), with the protein dissolved (conc. 0.5 mg/ml) in 50 mM Tris buffer at pH 7.3, in the range 20–95°C, at a speed of 1°C per minute. The samples and references were degassed immediately before use. C_p_ (cal/°C) was plotted against °C. Micro Cal LLC DSC software was used for data acquisition, analysis and data were corrected for buffer baseline prior to concentration normalization.

Time- dependent light scattering measurements of both wild type and V42M were done by monitoring the protein solution turbidity at 600 nm, using the spectrofluorimeter mentioned above, with 2.5 nm excitation and emission slits, at the constant temperature of 57°C, with the protein concentration of 0.1 mg/ml in 50 mM Tris buffer.

### Molecular Modeling and Dynamics Simulation

Models of the wild type HGSC and the V42M mutant were built separately utilizing the structure (PDB ID 2A5M) of the gamma S crystallin from *Mus musculus* template. The Swiss-Model workspace was used for this [29]. Structures obtained were soaked in ∼1200 molecules of water, and the charge of the ensemble adjusted to 0 using sodium ions. One cycle of minimization (200 iterations, steepest descent) was performed with the protein restrained; followed by another cycle (500 iterations, steepest descent) without any restraint. The structure was heated to 300 K over 40 ps (time step 1 fs), and finally minimized for 200 iterations using the adopted basis Newton-Ralphson method. These protocols were implemented in the CHARMM simulation environment, version c33b2 [30]. Structures were visualized using an Accelrys Discovery Studio system.

## Results

### The Mutant Precipitates Upon Standing

It was possible to recover both the wild type and the mutant proteins from the soluble fractions during the purification and isolation steps, suggesting that the solubility of V42M HGSC to be not severely compromised in comparison to that of the wild type; however, upon standing at 4°C, it starts precipitating, unlike the latter. This behavior is similar to that of some of the congenital cataract associated mutants of human γC- and γD-crystallins.

### V42M Exposes its trp to the Surface

**Figure 1:**
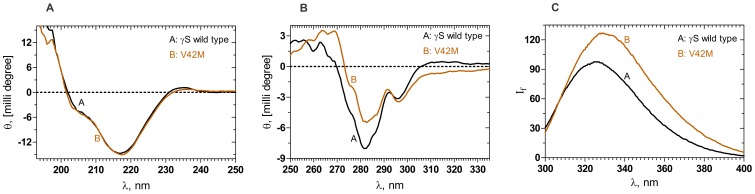
Mutation does not affect the back bone conformation but alters the tertiary fold of the protein. (A): Mutation does not affect the secondary structure of the protein. Far-UV CD spectra of wild type and mutant γS crystallin V42M. A (Black): γS Wild type; B (Brown):V42M. Protein concentration in each of the case was 10 µM (0.2 mg/ml) in 10 mM sodium phosphate buffer (pH 7.3), cell path length 2 mm, all spectra were recorded at 27°C, corrected for background buffer signal and each spectrum is an average of 3 independent runs.(B): Tertiary structure around the aromatic residues is slightly altered. Near UV CD spectra of wild type and mutant γS-crystallin V42M. A (Black): γS Wild type; B (Brown):V42M. Protein concentrations was 40 µM (0.8 mg/ml) in 100 mM sodium phosphate buffer, (pH 7.3), cell path length 10 mm and the other conditions of measurement were the same as above. (C): Mutation affects the environment around the trp residues. Intrinsic fluorescence of wild type and mutant γS crystallin V42M. I_f_: emission intensity in arbitrary units. A (Black): γS Wild type; B (Brown):V42M The protein concentrations used were 10 µM (0.2 mg/ml) in 100 mM sodium phosphate buffer, (pH 7.0), cell path length 3 mm, and spectra were recorded at 27°C, using an excitation wavelength of 295 nm, with 2.5 nm slits.

Figure 1A compares the far-UV circular dichroism (CD) spectra of wild type and V42M HGSC; there is no difference in the spectral profiles of the two proteins. The negative band at 218 nm, the shoulder at around 206 nm and the positive peak at 195 nm are in keeping with the predominantly β-sheet secondary structural fold of the chain and in accord with previous results [13], [14], [20]. The tertiary structural features were monitored using near-UV CD and fluorescence emission spectroscopy. Near-UV CD spectra (Figure 1B) reveal minor changes in the microenvironment around the aromatic residues of the mutant in comparison to the wild type. These are better visualized by fluorescence spectra than near UV-CD. HGSC has 8 phe residues, 12 tyr and 4 trp residues in its sequence. Of these, the trp residues exhibit the most notable fluorescence quantum yields, and hence most often studied by exciting at 295 nm and monitoring the emission in the 320–350 nm region. All the four trp residues are buried in the core of the native protein, and thus they emit in the 320–326 nm region, and also not accessible to ionic quenchers such as KI. When structural perturbation occurs, making one or more trp residues accessible to the surface (as upon denaturation), the emission wavelength maximum is red-shifted and the intensity increases. Figure 1C reveals that while the wild type emits at 326 nm with an intensity of 98 arbitrary units, the mutant emits at 327.5 nm (slight redshift) but with an enhanced intensity of 127 units. This 25% enhancement in the emission intensity in V42M suggests a somewhat higher exposure of the aromatic residue to the solvent. In addition, we found that while the ionic quencher KI had little effect on the wild type molecule (in accordance with earlier published on the bovine gamma S crystallin) [21]. The mutant suffered partial quenching (with a Stern- Volmer value K_SV_ = 0.88). The neutral quencher acrylamide too showed some slight difference between the wild type (K_SV = _1.12) and V42M (K_SV_ = 1.93).

**Figure 2:**
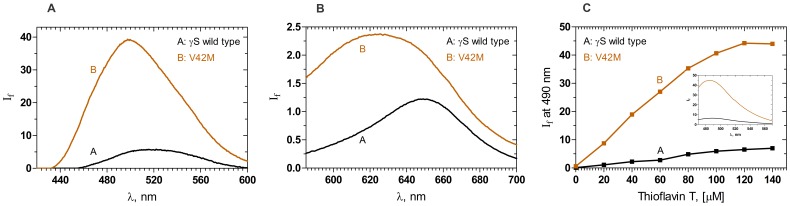
Mutation makes the protein aggregation-prone. (A): Mutation exposes nonpolar residues to the surface. Surface exposure of residues in the wild type and mutant γS crystallin V42M, monitored using bis-ANS as the extrinsic probe. A (Black): γS Wild type; B (Brown): V42M; λ_exc_: 390 nm, cell path length 3 mm, 2.5 nm slits. (B): Mutant readily self-aggregates. Aggregation tendencies of the wild type and mutant γS crystallin V42M, estimated using Nile Red as the extrinsic probe. A (Black): γS Wild type; B (Brown): V42M; λ_exc_: 540 nm, cell path length 3 mm, excitation emission slits 10 nm. (C): Aggregation appears amyloidogenic. Amyloid-type aggregation of wild type and mutant γS crystallin V42M, probed using Thioflavin-T. A (Black): γS Wild type; B (Brown): V42M; I_f_ of the probe at 490 was measured as a function of increasing concentration. cell path length 3 mm, excitation and emission slits 10 nm. Inset shows the actual spectra of Thioflavin (120 µM) when added to wild type (black) and mutant (brown). Protein concentration in each case was fixed at 5 µM (0.1 mg/ml) in 100 mM sodium phosphate for all these three experiments.

The extent of surface exposure is better monitored using the extrinsic reporters bis-ANS [22] and Nile Red [23], which display enhanced emission intensity upon binding to hydrophobic surfaces. Figure 2A shows that bis-ANS exhibits a 24 nm blue-shift in its emission maximum, and an eight-fold increase in its emission intensity upon binding to the mutant V42M than when it is bound to the wild type, indicating that V42M has a higher degree of surface hydrophobicity. The second probe, Nile Red, also known to be a sensitive detector of protein self- aggregation, showed a 16 nm blue-shift in emission (625.5 nm cf. 651 nm with wild type) and over twofold increase in its emission intensity upon binding to the mutant V42M (Figure 2B) than when bound to the wild type HGSC; this result too suggests a greater exposure of nonpolar residues to the solvent.

We next used the dye Thioflavin-T, which upon binding to an amyloid-type fibril-forming protein, displays an enhanced intensity in its emission band in the 470–570 nm region when excited at 444 nm [26]. Figure 2C shows the intensity of Thioflavin-T emission (monitored at 490 nm) is almost eight-fold higher with V42M than when it is bound to the wild type protein. The actual spectra of Thioflavin-T (120 µM) when added to wild type and when added to V42M are shown in Figure 2C as insets, to aid comparison. The behavior of V42M is thus similar to that of murine γB-crystallin mutant, also under normal physiological conditions [31], though further proof using FTIR and electron microscopy would be needed before concluding that the observed aggregates are indeed amyloid in nature.

### The V42M Mutant Displays a Three-state Conformational Transition

**Figure 3:**
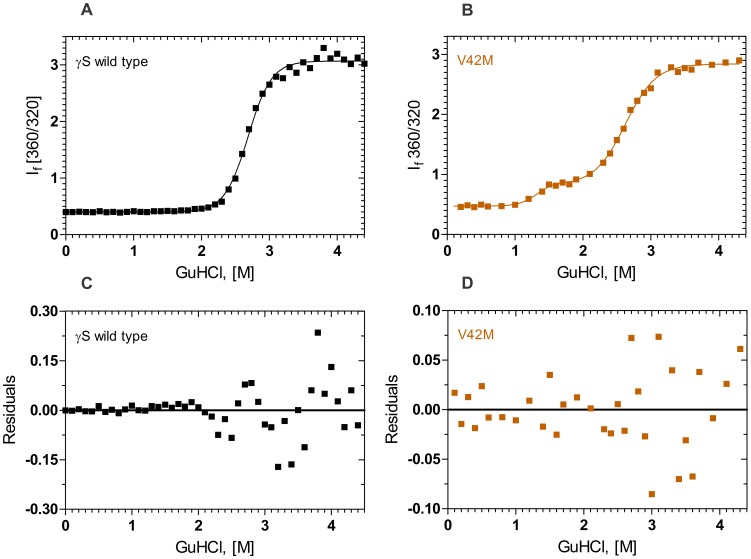
The mutant is structurally weaker than the wild type molecule. Guanidine hydrochloride induced denaturation of wild type and mutant γS crystallin V42M. Samples were excited at 295 nm and the relative emission intensity of the 360 nm band (of the denatured form) was compared to that of the 320 nm band (of the native protein) and monitored as a function of denaturant concentration. Solid line indicates the fitted data and solid blocks stand for raw data. Protein concentration in each sample was fixed at 0.2 mg/ml in 100 mM sodium phosphate, 1 mM EDTA and 5 mM DTT, cell path length 3 mm, and spectra were recorded at 27°C, using an excitation wavelength of 295 nm, with 2.5 nm slits. Residuals of wild type and mutant are also shown below the graphs.

Figures 3A and 3B show the results of the GuHCl induced denaturation of the two proteins, monitored using the fluorescence intensity ratio I_f_ 360/I_f_ 320. Both the wild type and V42M display a major unfolding transition between 2 to 3.5 M GuHCl concentration, with the midpoint of transition around 2.8 M GuHCl, consistent with earlier reports [20]. However, note that the mutant V42M displays an earlier transition, with its midpoint around 1.2 M GuHCl, indicating the population of a partially unfolded intermediate. The ΔG^0^ values of this first transition in V42M (Table 2) was estimated to be about 4.2 kcal mol^−1^, while that for the second transition was 6.8 kcal mol^−1^. In comparison, the wild type HGSC denatures at 2.8 M GuHCl, with a ΔG^0^ value of 9.4 kcal mol^−1^. The wild type molecule exhibits the classical two state behavior of unfolding, while the mutant has a three state transition; note however, that its second transition occurs at the same 2.8 M GuHCl as the wild type.

**Table 2:** Equilibrium unfolding parameters for wild type and V42M.

	Equilibrium transition 1		Equilibrium transition 2	
Protein	*C_m_*	Apparent ΔG^0^	*C_m_*	Apparent ΔG^0^
wild type			2.8	9.420±0.439 [Δ**G^0^_N-U_**]
V42M	1.2	4.201±0.610 [Δ**G^0^_N-I_**]	2.8	6.885±0.443 [Δ**G^0^_I-U_]**

[*C_m_* ] Transition midpoint in the units of M GuHCl.

[ΔG^0^] Free energy of unfolding in the absence of GuHCl in units of kcal mol^−1.^

N-U refers to native to unfolded transition, N-I native to intermediate and I-U intermediate to unfolded state.

### Mutation Decreases the Thermal Stability of HGSC

**Figure 4:**
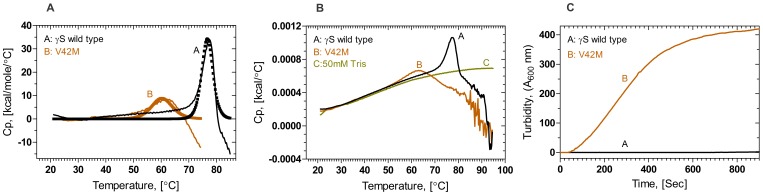
The mutant has less thermal stability and prone to aggregate faster than wild type. (A): Wild type (Black) and V42M (Brown) fitted curves were obtained from cursor initiative fitting procedures by normalized data. Solid line indicates the raw data and blocks stands for fitted data (B): Raw DSC thermograms of wild type (Black), V42M (Brown) and 50 mM Tris buffer (baseline, in olive green). Protein concentration of 0.5 mg/ml was used and heated in the range of 20–95°C with a rate of 1°C/min. (C): **Mutant starts scattering light when heated at 57°C while the wild type does not.** Time course of light scattering by the proteins at 600 nm light, using 2.5 nm excitation and emission slits. A protein concentration of 0.1 mg/ml in 50 mM Tris buffer was used. Wild type (Black) and V42M (Brown), cell path length 10 mm, and spectra were recorded at 27°C with 2.5 nm slits.

Figure 4A shows the results of the thermal unfolding of the two proteins, obtained using differential scanning calorimetry. Wild type HGSC shows the thermal transition at 76.7°C, while the mutant displays the transition far earlier, at 60.3°C, showing thereby that the mutant is weaker in stability. However, estimation of the enthalpies of unfolding was given up, since the proteins solutions began to turn cloudy and starting to precipitate just before, during and after the transition. This led to a poor goodness of fit (unacceptable χ^2^ values), thus allowing us only to report the Tm values.

In the calorimetry experiment, we found the mutant to progressively start forming a cloudy suspension with increasing temperature, even before its denaturation temperature of 60.3°C, while the wild type was clear until it reached its denaturation temperature of 76.7°C. This led us to monitor the time dependence of light scattering, i.e., turbidity at 600 nm, at the constant temperature of 57°C, for both wild type and V42M. This is shown in Figure 4C, where we note that while the wild type does not scatter light even after 800 s, the mutant does so quite rapidly and visibly to the naked eye, already by 400 s of incubation.

### Calcium Binding Propensity

**Figure 5:**
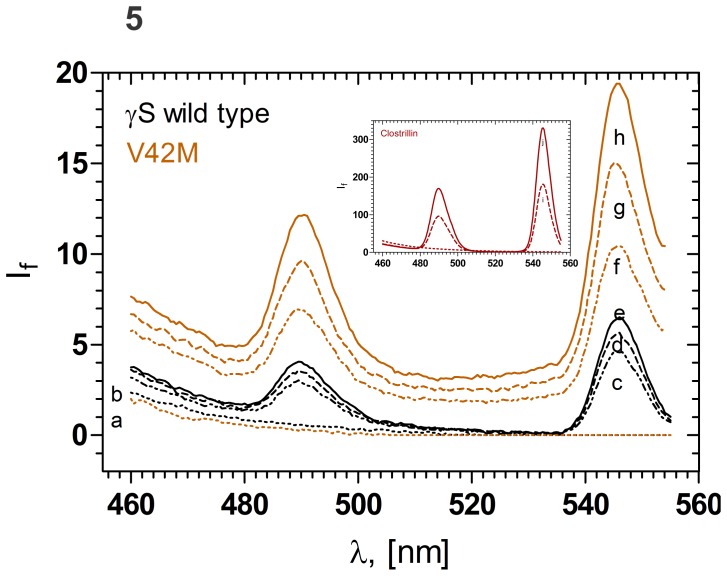
Comparison of the Calcium ion binding abilities of wild type and V42M γS-crystallin, monitored using the Ca^+2^-mimic Tb^+3^ ion. 5 µM (0.1 mg/ml) protein solutions were titrated with varying concentrations of TbCl_3_ and the fluorescence emission intensity plotted. Bottom-most line: protein alone, with no added TbCl_3_. Curves c, d and e: wild type protein with increasing amounts of TbCl_3_ added (300, 450 and 600 µM Tb^+3^); top three curves f, g and h: V42M mutant, with increasing amounts of added TbCl_3_ (300, 450 and 600 µM Tb^+3^); I and j (inset): clostrillin with 15 µM Tb^+3^ and 30 µM Tb^+3^, cell path length 10 mm, and spectra were recorded at 27°C, using an excitation wavelength of 285 nm, with 5.0 nm slits. Inset:Tb^+3^ binding ability of a “standard” Ca^+2^ binding crystallin family of protein, clostrillin [ref. 32,33], shown for comparision; note the scale change in intensity.

Coming to the issue of calcium ion binding by the crystallins, it is now reported that numerous proteins of the βγ-crystallin family carry the residue sequence motifs N/D-N/D-X(1)-X(2)-S/T-S, to which calcium ions bind in a ‘double clamp’ format [32], [33]. Analyses of the sequence of HGSC reveals that one double clamp motif to be present (the residue sequence 34–39 LSRCNS and the sequence 77–82 NDRLSS, both in the N-td, and both in the wild type and the mutant molecule). We hence undertook to study whether the wild type and V42M HGSC display any difference in their Ca^2+^ binding properties, by using the Ca^2+^ mimic lanthanide trivalent ion probe Tb^3+^, which binds at Ca^2+^ binding sites, and displays enhanced luminescence in the visible region of the spectrum, through fluorescence resonance energy transfer [24], [25]. As Figure 5 shows, Tb^3+^ binds to both the wild type and the mutant with about the same spectral features, suggesting that both proteins bind Ca^2+^ in comparable manner. As a standard comparison, we used clostrillin, which is known to bind Ca^2+^ with a Kd value of 5 µM [32], while the γ-crystallins bind Ca^2+^ weaker, with a Kd value of 90 µM [25]. It is still not clear why the mutant has a higher set of If values than the wild type. Two possible reasons could be: (a) the aromatic residue trp 47 in the mutant is more exposed and accessible than in the wild type, thus making energy transfer to the Tb^3+^ more efficient, and (b) the relatively electron-rich sulfur atom in the side chain of M42, with its high electronegativity might also allow a higher degree of interaction with Tb^3+^.

## Discussion

### Comparison of the Behavior of V42M with Other Mutants of HGSC

γS-crystallin is the most highly conserved member of the crystallin family in evolution [34] and retains a very high sequence homology (>90%) among mammals. It is expressed abundantly in the mammalian lens nucleus and cortex. Four mutations in HGSC have so far been reported to be associated with congenital cataracts. These are (i) G18V, leading to progressive cortical cataract [9], (ii) D26G, leading to a Coppock-type phenotype [10], (iii) S39C, associated with microcornea and cataract [11], and (iv) V42M, the subject of our study, which leads to autosomal dominant bilateral cataract [12]. Of the four, V42M is the severest in phenotype, namely, an opalescent lens with the central nuclear region denser than the periphery. While protein structural analysis has been done for the mutant G18V [13], [14], [35], [36], and we report here our studies on V42M, detailed analysis of D26G and S39C are yet to be done.

As with other γ-crystallins, HGSC too is a two-domain protein. The structural details of the native form and denaturation profiles of the isolated N-terminal domain (N-td, residues 1–86), isolated C-terminal domain (C-td, residues 93–177), as well as the intact, full-length two domain molecule (wild type) HGSC have been described in detail by Mills et al. [20]. They have shown that the N-td folds slightly differently (in its secondary structure) and unfolds easier (unfolds at 69°C, and at 1.7 M GuHCl, cf. 75°C and 2.3 M GuHCl for the C-td, and 74°C and 2.3 M GuHCl for the intact protein) than the C-td or the full length HGSC, showing that the N-td is inherently less stable than the C-td or the whole molecule. Given this, it is noteworthy that all the four congenital cataract-associated mutations in HGSC are located in the weaker N-td region and at least two of these, namely, G18V and V42M, weaken the structure of the whole molecule. In the case of G18V, replacement of the highly conserved glycine (which is required to stabilize the folded hairpin of the first Greek Key motif) by the bulkier valine destabilizes the fold somewhat [13], [37] and while the valine is still buried, the mutation appears to expose cys residues thus allowing for disulfide-mediated aggregation as well [35], [36]. G18V is not only less stable than the wild type, but also shows a three-state denaturation profile, with a partially unfolded state at low GuHCl concentrations, and the second transition occurring very close to that of the wild type, suggesting that the stability of the C-td is not affected much due to the mutation [13].

### Molecular Modeling and Dynamics Analysis of V42M Reveal Distortion of the Compact β-sheet Core of the Greek Key Topology

**Figure 6:**
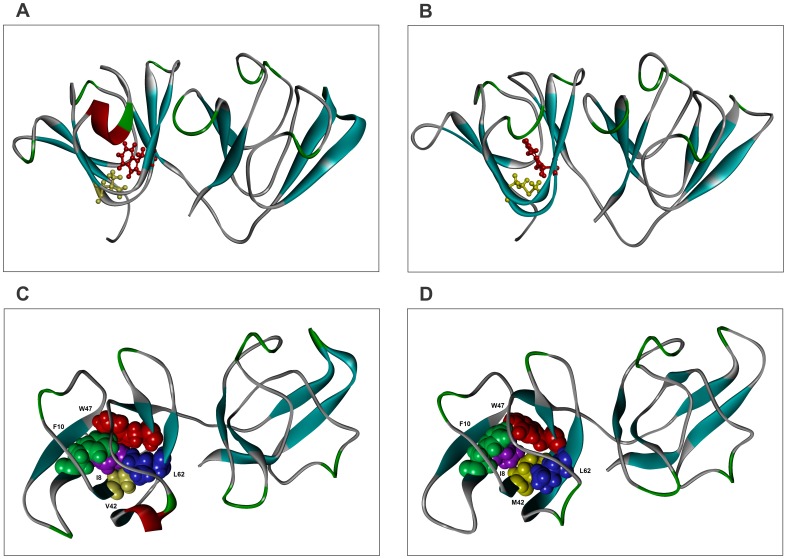
V42M leads to a distortion in the compactness of the N-terminal fold. Structures A and B show top views of the homology ball-and stick models of wild type (structure A) and V42M mutant (structure B) of HGSC. Structures C and D show the residues in the core of the N-terminal domain of the protein, and in the vicinity of V42 (and M42) as space-filling CPK models (I8, violet; F10, green; W47, red; L62, blue and V/M42, yellow). The rest of the protein is represented as a ribbon. The tighter packing of these residues is evident in the wild type molecule (structure C), which is loosened in the M42 mutant (structure D). Note that the C-terminal domain is essentially unaffected by the mutation.

Turning to our molecule, V42M, bioinformatic analysis to test the effect of the V42M substitution, using PolyPhen [38], predicted a high probability of distortion of the structure. To examine the structural effects of V42M substitution, we built homology models using murine γ-S crystallin as template, with which the human protein shares an 89.8% identity (see Figures 6A and 6B for the model structures of the wild type and mutant proteins). Analysis of the wild type HGSC showed the residue V42 to be in close proximity with I8, F10, W47, and L62, making a compact nonpolar core in the N-terminal domain. It is worth noting that in addition to V42, the residues I8, F10, W47 and L62 are also conserved not only among the γ-crystallins of humans, but also in dogs, rabbits, guinea pigs and mice. (In human γD-crystallin, F10 is replaced by L10, an equally hydrophobic residue of comparable van der Waals radius and non-polar surface area). It would thus appear that these residues are vital for the compact, nonpolar core structure of this domain of these molecules. An analysis of the mutant V42M HGSC showed that fitting the larger methionine residue into the confines of this core causes a strain on the integrity of the structure. Figures 6C & 6D reveal this strain and ‘opening up’ of the compact core. Preliminary molecular dynamics analysis has suggested this strain is not relieved in the course of annealing the molecule from low temperature to ambient. This *in silico* analysis suggests that this strain and consequent opening up causes a distortion of the compact β-sheet compact core topology of the N-terminal domain of the molecule, though the canonical Greek key fold itself is strained but not lost. Note from a comparison of the figures, too, that the C-terminal domain does not appear to have been affected by the mutation.

One readily noticeable effect of this structural strain is seen in the alteration in the spatial disposition of W47 in the mutant vis-à-vis the wild type molecule (see Figures 6A and 6B). This change is reflected both in the fluorescence behavior of the mutant (Figures 1C, 2A and 2B), and its denaturation curves. The first transition of the mutant (Figure 3B) occurs even earlier than that of the isolated N-td of the wild type (1.2 M GuHCl vs 1.7 M GuHCl in the latter). However, the second transition occurs at about the same concentration of GuHCl as the wild type, suggesting (as in the case of G18V) that the stability of the C-td region is not affected by the mutation in the N-td, and stays the same as in wild type (The C-td portions in Figures 6 seem to confirm this). These results are in comparison to those of both Mills et al. [20] and Wenk et al. [39], which show that HGSC, as well as its isolated N-td and isolated C-td all follow a two-state transition in their chemical denaturation profile. In contrast, our results here show how the single replacement of V by M changes the stability of the molecule. The mutant’s free energy of unfolding (ΔG°_1_ = 4.2 kcal/mol, and ΔG°_2_ = 6.8 kcal/mol) is lower than that of the wild type (ΔG°_1_ = 9.4 kcal/mol). These results suggest that the mutation in the N-terminal domain makes the mutant molecule follow a three-state conformational transition. Similarly, upon heating the protein (thermal denaturation), the mutant denatures at 64°C, suggesting a weaker folding than G18V (60.3°C) [13], or the wild type (76.7°C).

Gamma crystallin mutants of this kind tend to invariably aggregate. That V42M does so while the wild type does not is shown by (a) the observation that upon standing at 4°C, a solution of 0.2 mg/ml of the mutant precipitates while that of the wild type does not, (b) the differences in the temperature-dependent scattering of light between V42M and the wild type (Figure 4C). In addition, lens crystallins have been reported to tend to form amyloid type aggregates, particularly when their structure is altered [31], [40]. As mentioned earlier, it is yet to be confirmed whether the aggregates formed by V42M are amyloid type or not. We have initiated such a study with a set of human γ-crystallin mutants.

The function of the Greek key motif in the βγ-crystallins is yet to be fully understood. They are important in folding the two domains (N-td and C-td) of the proteins and the inter-domain interactions that make the molecule compact and globular, allowing dense packing of the protein molecules, offering short range interactions (almost liquid-like) to dominate, thus remarkably reducing light scattering and offering transparency to the lens [2], [41], [42]. This tight packing is so fine-tuned that, in the present case, even the ‘simple’ replacement of a valine residue (van der Waals volume 105 A^3^, average volume of buried residue 142 A^3^, and accessible surface area 117 A^2^) by methionine (van der Waals volume 135 A^3^, average volume of buried residue 171 A^3^, and accessible surface area 167 A^2^) [43] is not tolerated, leading to self-aggregation. It appears likely that any mutation which distorts even one of the Greek key folds in the βγ-crystallins would perturb this compactness, intermolecular packing and consequently the transparency of the regions of the lens where the mutant protein is abundantly present.
